# ﻿*Euonymuspushpagiriensis* (Celastraceae), a new species from the central Western Ghats of Karnataka, India

**DOI:** 10.3897/phytokeys.253.138418

**Published:** 2025-02-25

**Authors:** Navendu V. Page, Tejas U. Thackeray

**Affiliations:** 1 Thackeray Wildlife Foundation, Mumbai, 400020, India Thackeray Wildlife Foundation Mumbai India

**Keywords:** Endemic, *Euonymus*, India, Kodagu, Western Ghats

## Abstract

*Euonymuspushpagiriensis***sp. nov.**, an understorey tree species from the montane (Shola) forests of Kodagu District of Karnataka is described and illustrated. The species is morphologically distinct from all other species of *Euonymus* reported from the Western Ghats and the rest of India, in having sub-sessile, ovate leaves with rounded to sub-cordate leaf base. This species is, so far, known from the Kodagu District of the State of Karnataka.

## ﻿Introduction

The genus *Euonymus* is represented by roughly 145 species of woody plants. It is distributed in the tropical and temperate regions of the Northern Hemisphere, mainly across North America, Europe, South and South East Asia. Its distribution range, however, also extends into some of the tropical regions of Southern Hemisphere including Madagascar, Papua New Guinea and north-eastern Australia (POWO 2024). The first comprehensive revision of the genus *Euonymus* was carried out by Blakelock (1951) and more recently by Ma (2001). The genus is characterised by woody and mostly erect (rarely scandent or trailing) habit, opposite or sub-opposite leaves, four or five-merous flowers produced in axillary (or rarely cauliflorous) inflorescences, stamens inserted on the periphery of the disc, loculicidal dehiscent capsules and arillate seeds (Li et al. 2024).

In India, the genus is represented by ca. 30 species (Ramamurthy 2000; Murugan and Manickam 2005; Murugan and Manickam 2006), of which ca. 20 species are concentrated in the north east India, a few of which also extending their distribution to the Western Himalayas, while two species are distributed in Andaman and Nicobar Islands. Eight species of *Euonymus* are reported from south India, all of which are distributed in the tropical wet evergreen forests of the Western Ghats and exhibit geographic distributions that are restricted to this region, with the exception of *Euonymuslaxiflorus*, which shows disjunct distribution in southern China.

## ﻿Materials and methods

From year 2010 to 2014, plot-based inventory of woody flora of the evergreen forests of the Western Ghats, was carried out as part of the PhD study of the first author. Details about the sampling methodology and sampling locations are presented in Page and Shanker (2018) and Page and Shanker (2020). During this period, while compiling an inventory of the Central Western Ghats in the Kodagu District of Karnataka, we found a species of *Euonymus* that was morphologically distinct from the hitherto described species from the Western Ghats. To confirm the taxonomic novelty of the species, we compared its morphological characters with the rest of the species reported from Indo-China, based on the literature (Gamble 1915; Blakelock 1951; Ramamurthy 2000; Ma 2001; Murugan and Manickam 2005; Murugan and Manickam 2006; Li et al. 2024). We found that the population from Kodagu is morphologically distinct from all the species described so far, based on the leaf and inflorescence characters. It is, therefore, described here as a new species. The description provided is based on field observations of four individuals from two different locations. Detailed colour plates of the new species, as well as its morphologically most closely related species, are provided to aid identification in the field.

## ﻿Taxonomic treatment

### ﻿ Euonymus pushpagiriensis

Taxon classificationPlantaeCelastralesCelastraceae

N.V.Page & T.U.Thackeray sp. nov.

1DC791C6-E0F9-59C0-A962-39E2CE8E41E5

urn:lsid:ipni.org:names:77357224-1

Figs 1 , 2 , 3 , 4

#### Type.

India. • Karnataka State, Kodagu District, Mandalpatti, alt. 1240 m, 12°32'23.02"N, 75°42'10.66"E, 28 April 2013 (fl.), *N.V.Page 28413* (holotype: MH!; isotypes MH!, JCB!)

#### Diagnosis.

*Euonymuspushpagiriensis* can be distinguished from other species of *Euonymus* from the Western Ghats based on its sub-sessile leaves (petiole ca. 1 mm long) and ovate lamina with rounded to sub-cordate base. In contrast, all the other species of *Euonymus* from the Western Ghats of south India exhibit leaves with a distinct petiole (greater than or equal to 3 mm length) and elliptic, rarely ovate lamina with obtuse, acute or cuneate base.

**Figure 1.**
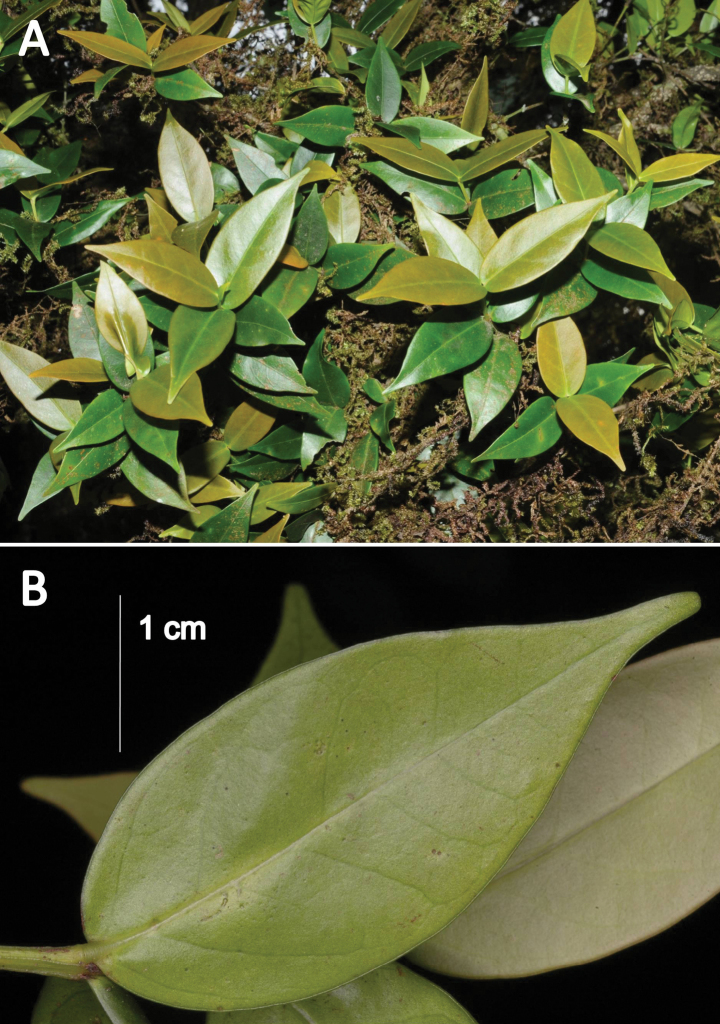
*Euonymuspushpagiriensis***A** branches with light brown young and dark green mature leaves **B** abaxial surface of a leaf with sub-sessile nature, rounded leaf base, entire leaf margin, inconspicuous nerves and acuminate leaf apex with obtuse tips. Note the ducurrent nature of petiole which forms narrow wings extending along the inter-node. Photographs by Navendu Page.

**Figure 2.**
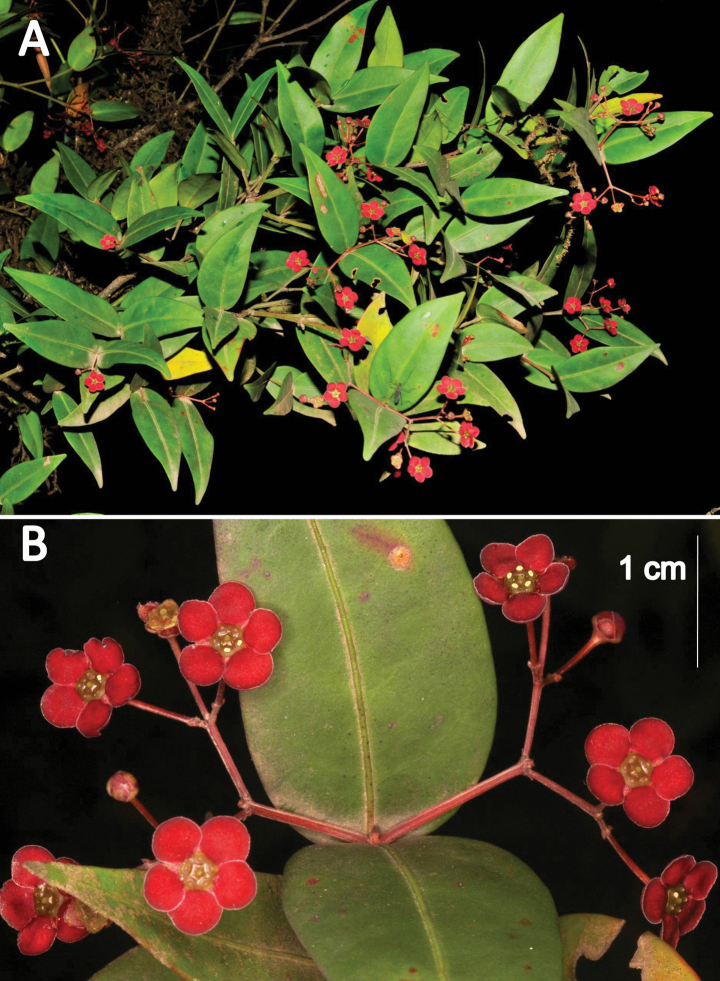
*Euonymuspushpagiriensis***A** branches with inflorescences borne in the axils of terminal pairs of leaves **B** dichasial inflorescences showing the four angled primary and secondary peduncles. Photographs by Navendu Page.

**Figure 3.**
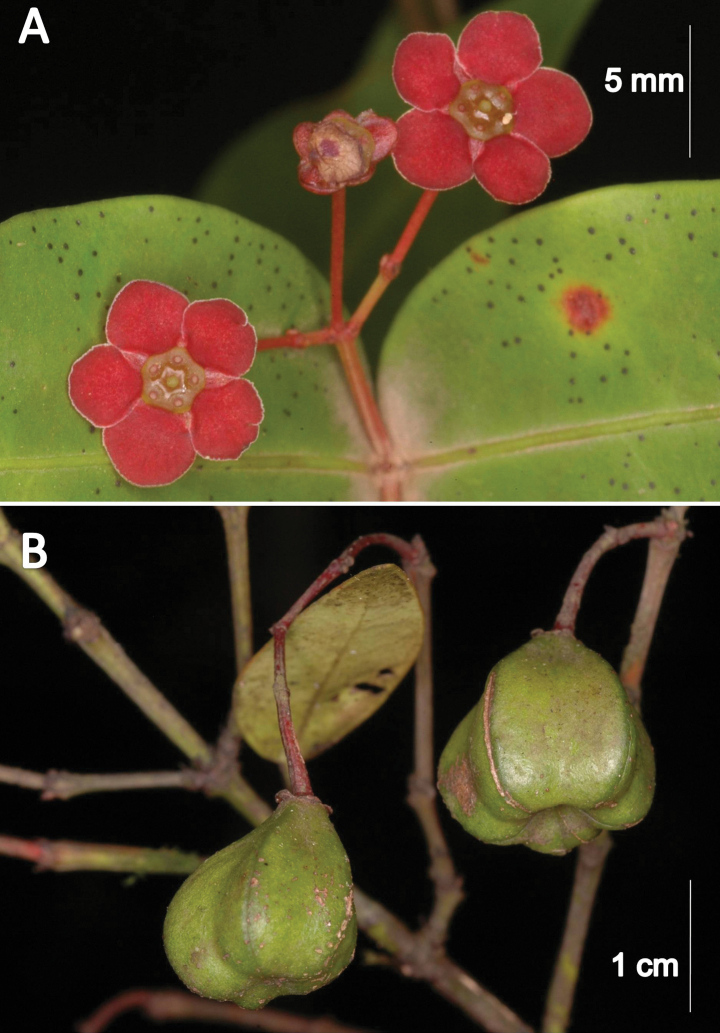
*Euonymuspushpagiriensis***A** five-merous flowers with pentagonal disc, five angled ovary and stamens **B** immature capsule. Photographs by Navendu Page.

**Figure 4.**
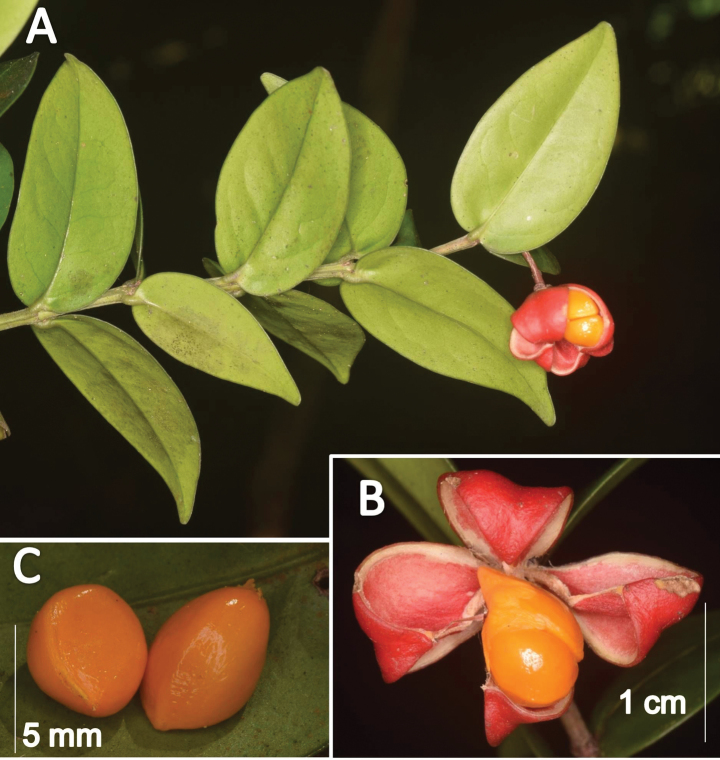
*Euonymuspushpagiriensis***A** branch with mature dehisced capsule **B** dehisced capsule with two arillate seeds in a cell **C** seeds with aril removed. Photographs by Navendu Page.

**Figure 5.**
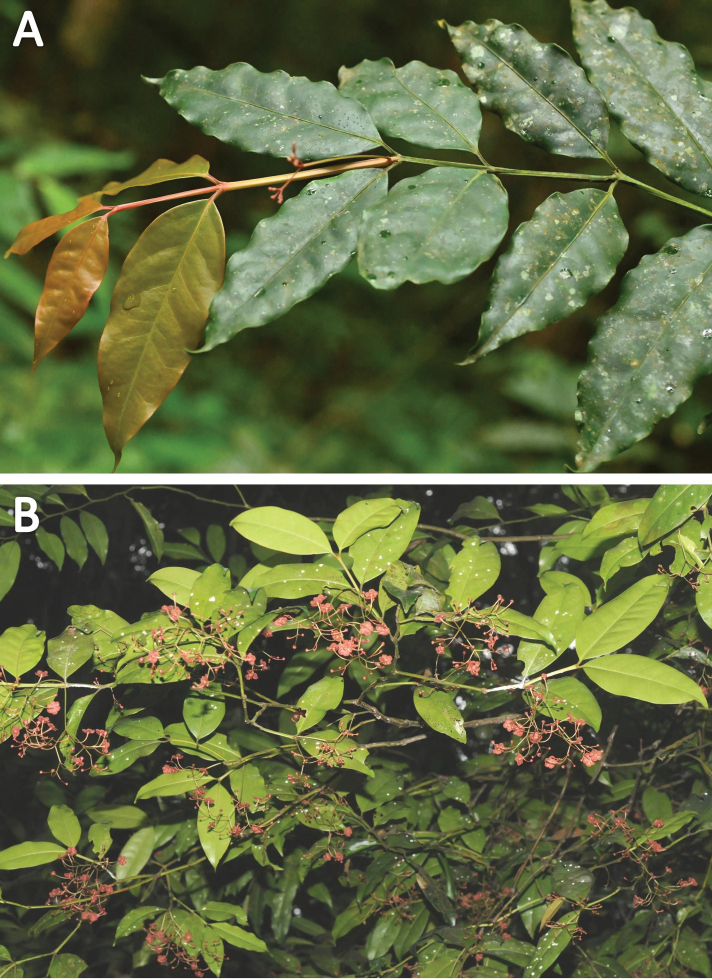
*Euonymusangulatus***A** dorsal view of branch with young inflorescences **B** ventral view of branches with mature inflorescences. Photographs by Navendu Page.

**Figure 6.**
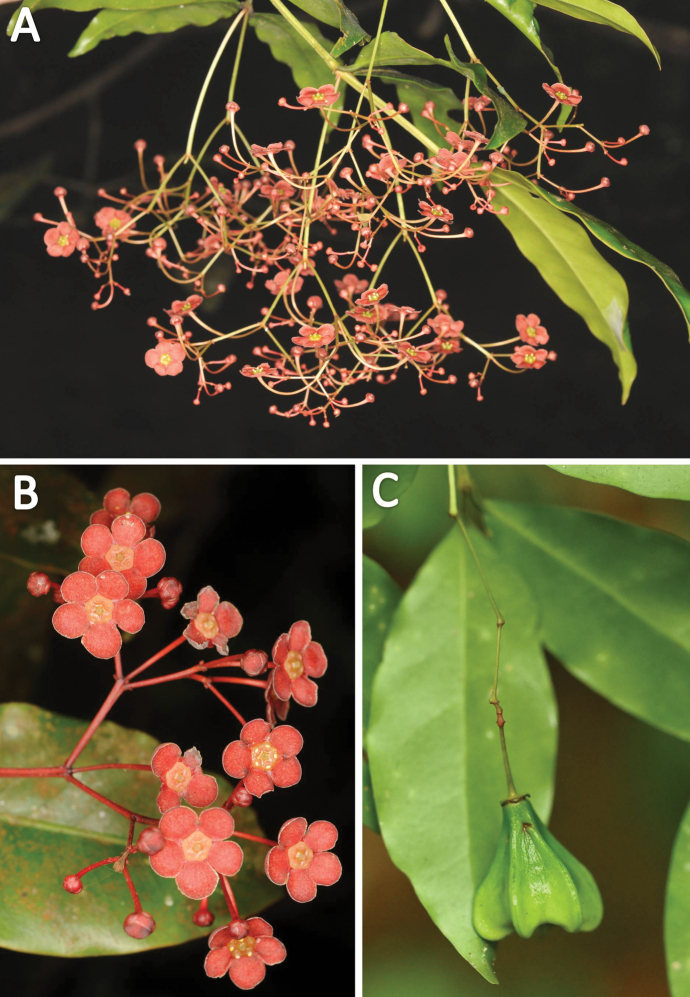
*Euonymusangulatus***A** inflorescences showing the drooping or pendulous nature **B** flowers **C** fruit. Photographs by Navendu Page.

*E.pushpagiriensis* (Figs 1–4) is morphological closely related to *E.angulatus* (Figs 5, 6) in having leaf margins entire and young branches and peduncles four angled. It can be easily distinguished from the latter, based on petiole length which are less than or equal to 1 mm (as opposed to petioles more than or equal to 3 mm in *E.angulatus*), rounded to sub-cordate leaf base (leaf base acute or obtuse, but never rounded in *E.angulatus*) and inflorescences borne in the axils of terminal pair of leaves (inflorescences extra-axillary or from the axils of older leaves from the last year’s growth in *E.angulatus*). Morphological differences between these two species are provided in Table 1.

**Table 1.** Key characters that distinguish *Euonymuspushpagiriensis* sp. nov. from its morphologically closely-related species *Euonymusangulatus*.

Character	*E.angulatus*	*M.pushpagiriensis*
Petiole	≥ 3 mm long	≤ 1 mm long
Leaf lamina shape	Elliptic	Ovate- lanceolate
Leaf base	Acute or obtuse, never rounded or sub-cordate	Rounded or sub-cordate
Leaf margin	Usually entire, rarely with a few serrations, not revolute	Always entire, obscurely revolute
Inflorescence	Borne from axils of older leaves from the last year’s growth	Borne only in the axils of terminal pair of leaves
Peduncle	Usually drooping, flexuose	Erect, rigid
Pedicel	Curved upwards	Erect, not curved
Capsule	Turbinate, deeply 5-lobed, attenuate at the base	Obovoid, with 5 angles and shallow grooves
Distribution	Mid to high (1000–1600 m) elevation forests on the windward slopes of Nilgiri and south Western Ghats of Kerala State	High (1200–1550 m) elevation forests of Pushpagiri mountain range in the Kodagu District of Karnataka State

#### Description.

Understorey, evergreen shrubs or small trees, up to 3 m tall. Twigs glabrous, narrowly 4-winged, quadrangular in cross section, bud scales minute, usually persistent at nodes. ***Leaves*** opposite, lamina glabrous, thick, coriaceous, 3.5–5.5 × 1.5–2.7 cm, ovate; base rounded or subcordate; apex acute to acuminate, rounded at the tip; margin entire, obscurely revolute; petiole stout, ca. 1 mm, decurrent in to the wings of the stem; mid-vein distinctly raised adaxially, as well as abaxially; lateral nerves inconspicuous, 4–7 on each side of mid-vein, looping at the margin. ***Inflorescences*** compound dichasium, solitary, borne in the axils of the terminal leaves; primary peduncles four angled, 1.3–1.7 cm long; secondary peduncles up to 0.5 cm long; bracts at the base of primary and secondary peduncles, lanceolate,1–1.5 mm long. ***Flowers*** 6–15 per inflorescence, 5-merous, ca. 7 mm in diameter; sepals subequal, subround, 1–1.5 × 0.5–1 mm, margin erose; petals subround, 2–2.5 × 2.5–3 mm, red, margin white, finely crenulate; disc 5-lobed, ca. 2.5 mm in diameter; stamens 5, inserted in the middle part of the lobes of the disc, filaments subsessile; ovaries superior, 5-angled, stigmas round, short; ovules 2 per cell. ***Capsules*** obovoid, with 5 angles and shallowly grooved, apex concave, 1.4–1.6 × 1.1–1.3 cm, opening into 5-lobes at maturity. ***Seeds*** (1) 2 in each cell, ellipsoid, 5–6 × 4–5 mm, orange, partially covered by orange arils at base.

#### Additional specimens examined.

India. • Karnataka State: Kodagu District, Pushpagiri Peak, Pushpagiri Wildlife Sanctuary 12°39'42"N, 75°41'05"E, alt. 1550 m, 29 xii 2015, *N.V.Page 291215* (JCB).

#### Distribution.

*Euonymuspushpagiriensis* is so far known from two localities and is endemic to the Kodagu District of Karnataka.

#### Ecology.

The species is distributed in the understorey or the edge of montane evergreen ‘Shola’ forests between 1200 to 1550 m elevation. The species was found to be growing in association with *Nothopegia* sp., *Actephilaexcelsa*, *Memecylon* sp. and *Syzygiumlanceolatum*, amongst others. The species on both occasions was observed growing at the crest of the west-facing slopes of the Western Ghats.

#### Phenology.

*Euonymuspushpagiriensis* produces young leaves in the month of January. Flowering was observed from late April to May while the fruiting period starts from June and the fruits mature in the months of December and January.

#### Etymology.

The specific epithet refers to the type locality of species – Pushpagiri which is the name of the second highest peak in Kodagu District and the fourth highest peak in the State of Karnataka and also the name of the Wildlife Sanctuary in which the peak the located. The species is, so far, known only from Pushpagiri Wildlife Sanctuary and its adjacent areas.

#### Provisional conservation status.

The species is currently known from two locations within the Kodagu District of Karnataka State. The Area of Occupancy (AOO), as per the IUCN Red List guidelines, is estimated to be 8 km^2^. Based on the geographic range (Criteria B2), the species qualifies for the Critically Endangered category. However, the species does not meet two of the three conditions required for to qualify for the threatened category. It satisfies only the condition (a) which is that of the number of locations being less than five. There is no evidence to suggest a continuous decline (condition b) or extreme fluctuations (condition c) in range size or number of locations. Hence, *Euonymuspushpagiriensis* is provisionally assigned ‘Near Threatened’ category.

## Supplementary Material

XML Treatment for Euonymus pushpagiriensis

## ﻿Additional information

### Author ORCIDs

Navendu V. Page https://orcid.org/0000-0002-9413-7571

Tejas U. Thackeray https://orcid.org/0000-0002-9981-8763

### Data availability

All of the data that support the findings of this study are available in the main text.

## References

[B1] Blakelock RA (1951) A Synopsis of the Genus *Euonymus* L. Kew Bulletin 6(2): 210–290. 10.2307/4120601

[B2] Gamble JS (1915) Celastraceae. Flora of the Presidency of Madras, Vol 1 Ranunculaceae – Caprifoliaceae. Adlard & Son, London, 145–152. 10.5962/bhl.title.21628

[B3] Li X Wang S-L Wu W-H Li M Quin Y-H Peng D-R Luo K-W (2024) *Euonymusfangdingianus* (Celastraceae), a new cauliflorous species from southwestern China and northern Vietnam. Nordic Journal of Botany 12: e04514. 10.1111/njb.04514

[B4] Ma JS (2001) A revision of Euonymus (Celastraceae). Thaiszia Journal of Botany 11: 1–264.

[B5] Murugan C Manickam VS (2005) *Euonymuskanyakumariensis* – A new species of Celastraceae from India. Journal of the Bombay Natural History Society 102(2): 198–200. 10.54207/bsmps1000-2006-F7W038

[B6] Murugan C Manickam VS (2006) *Euonymusbarberi* – A new species of Celastraceae from Agasthiyamalai, India. Indian Journal of Forestry 29(2): 199–200. 10.54207/bsmps1000-2006-F7W038

[B7] Page NV Shanker K (2018) Environment and dispersal influence changes in species composition at different scales in woody plants of the Western Ghats, India. Journal of Vegetation Science 29: 74–83. 10.1111/jvs.12586

[B8] Page NV Shanker K (2020) Climatic stability drives latitudinal trends in range size and richness of woody plants in the Western Ghats, India. PLoS ONE 15(7): 1–23. 10.1371/journal.pone.0235733 PMC7365598 32673330

[B9] POWO (2024) Plants of the World Online. Facilitated by the Royal Botanic Gardens, Kew. https://powo.science.kew.org/ [Retrieved 08 September 2024]

[B10] RamamurthyK (2000) Celastraceae. In: Singh NP Vohra JN Hajra PK Singh DK (Eds) Flora of India Vol 5 Olacaceae – Connaraceae.Botanical Survey of India, Calcutta, 1–577.

